# Physical activity and relaxation in the work setting to reduce the need for recovery: what works for whom?

**DOI:** 10.1186/s12889-016-3457-3

**Published:** 2016-08-24

**Authors:** Margriet A. G. Formanoy, Elise Dusseldorp, Jennifer K. Coffeng, Iven Van Mechelen, Cecile R. L. Boot, Ingrid J. M. Hendriksen, Erwin C. P. M. Tak

**Affiliations:** 1 Netherlands Organisation for Applied Scientific Research TNO, Schipholweg 77, Leiden, The Netherlands; 2 Department of Psychology, Catholic University of Leuven, Tiensestraat 102, Leuven, Belgium; 3 Methodology and Statistics, Institute of Psychology, Leiden University, Wassenaarseweg 52, Leiden, The Netherlands; 4 Department of Public and Occupational Health, the EMGO+ Institute for Health and Care Research, VU University Medical Center, Amsterdam, The Netherlands; 5 Body@Work, Research Center Physical Activity, Work and Health, TNO- VU/VUmc, VU University Medical Center, Amsterdam, The Netherlands

**Keywords:** Motivational interviewing, Social environmental intervention, Environmental modifications, Physical activity, Worksite health promotion, Relaxation program, Need for recovery, Work, QUINT

## Abstract

**Background:**

To recover from work stress, a worksite health program aimed at improving physical activity and relaxation may be valuable. However, not every program is effective for all participants, as would be expected within a “one size fits all” approach. The effectiveness of how the program is delivered may differ across individuals. The aim of this study was to identify subgroups for whom one intervention may be better suited than another by using a new method called QUalitative INteraction Trees (QUINT).

**Methods:**

Data were used from the “Be Active & Relax” study, in which 329 office workers participated. Two delivery modes of a worksite health program were given, a social environmental intervention (group motivational interviewing delivered by team leaders) and a physical environmental intervention (environmental modifications). The main outcome was change in Need for Recovery (NFR) from baseline to 12 month follow-up. The QUINT method was used to identify subgroups that benefitted more from either type of delivery mode, by incorporating moderator variables concerning sociodemographic, health, home, and work-related characteristics of the participants.

**Results:**

The mean improvement in NFR of younger office workers in the social environmental intervention group was significantly higher than younger office workers who did not receive the social environmental intervention (10.52; 95 % CI: 4.12, 16.92). Furthermore, the mean improvement in NFR of older office workers in the social environmental intervention group was significantly lower than older office workers who did not receive the social environmental intervention ( −10.65; 95 % CI: −19.35, −1.96). The results for the physical environmental intervention indicated that the mean improvement in NFR of office workers (regardless of age) who worked fewer hours overtime was significantly higher when they had received the physical environmental intervention than when they had not received this type of intervention (7.40; 95 % CI: 0.99, 13.81). Finally, for office workers who worked more hours overtime there was no effect of the physical environmental intervention.

**Conclusions:**

The results suggest that a social environmental intervention might be more beneficial for younger workers, and a physical environmental intervention might be more beneficial for employees with a few hours overtime to reduce the NFR.

**Trial registration:**

NTR2553

## Background

Work stress and work-related health problems are a major problem in modern organizations [1]. When chronically exposed to high levels of psychological job demands, job variety, little autonomy or support from others, it may lead to increased stress levels as well as health problems [1]. Work-related stress can cause substantial economic costs due to lost productivity and absenteeism [2]. A 2010 European survey (*n* = 21,703) showed that 28 % of the workers experienced work-related stress [3].

An early precursor for work-related health problems is a higher Need for Recovery (NFR; [4]). NFR is described as the need to recuperate and unwind from work-induced effort and represents the short-term workload effects of a day at work [5]. It can be seen as an intermediate variable between psychosocial work characteristics and work-related health problems [6, 7].

Several health behavior strategies were studied to reduce work stress and work-related health problems, and/or NFR, among which physical activity and relaxation. Evidence was found that physical activity is valuable in unwinding from work [8, 9]. Sufficient physical activity, that is, three to five times a week for 15 to 45 min, resulted in lower work stress [10], reduced absenteeism [11], and improved job satisfaction [12]. In addition, participating in relaxation activities diverted the mind from work and improved self-esteem-feelings that are essential for recovery [13]. Not taking enough time to relax increased the NFR and was associated with exhaustion [14]. Furthermore, within a group of service employees, enjoyable and restful within workday breaks improved their need for recovery [15].

However, some studies did not succeed in improving the need for recovery by a worksite health program [16] or the effect did not sustain in the long-term [17]. One possible cause of this phenomenon could be that the studies focused on the effect(s) of a worksite health program for all workers included in the study (i.e., the treatment main effect). However, the question is whether the assumption that a program is equally effective for all workers is realistic. It may be that a “one size fits all” approach is not suitable in this case. In other words, the effectiveness of an intervention may differ across individuals. For example, Taris et al. [18] suggested that working overtime on a recurrent character may influence the results of workplace health interventions in two different ways. First, as a result of working overtime, less time is available for recovery. Second, people who work overtime recurrently are prone to make “unhealthy choices” concerning lifestyle, such as smoking and consuming high levels of alcohol. By incorporating (some of) these moderator variables into the design of the study, one can target the right population.

Therefore, it might be very useful to define subgroups that may benefit more from a certain worksite health program. The present study focuses on the identification of such subgroups using data from the “Be Active & Relax” study.

The worksite health program “Be Active & Relax” was developed to reduce the NFR in office workers [19] via the increase of daily physical activity and relaxation. The effectiveness of the intervention was investigated in a trial using a 2 × 2 factorial design. The design factors were two different strategies of delivering the “Be Active & Relax” program, in other words, two delivery modes. One delivery mode was a “social environmental intervention”, which consisted of a counseling style that focused on behavioral change in groups and was derived from motivational interviewing at the individual level [20]. The other delivery mode was a “physical environmental intervention”, which consisted of encouraging physical activity and relaxing behavior by making changes in the physical environment.

The results of the “Be Active & Relax” project showed that none of the delivery modes were effective in reducing the NFR [17]. Therefore, it was not recommended to implement the current interventions because the NFR did not significantly differ as a result of applying either delivery mode. And although some significant effects were found on work-related outcomes, these were only small (e.g., contextual performance, dedication, task performance, and absorption [21]).

To identify subgroups for whom one intervention might be better suited than another, several statistical methods are available. Based on the characteristics of the participants measured at baseline (so called moderator variables), the subgroups can be determined a priori (e.g., based on previous literature; also called confirmatory analysis) or a posteriori, that is, induced from the data (also called exploratory analysis). The classical approaches to identify subgroups are analysis of variance with paired comparisons, and multiple regression analysis with pre-defined interaction effects. A disadvantage of these methods is that they can handle only a small number of moderator variables. For the situation of many potential moderator variables that might interact with the treatment variable and no clear a priori hypotheses, several new methods were developed in the last decade based on recursive partitioning (see [22] for an enumeration), among which QUalitative INteraction Trees (QUINT) [23, 24]. These new methods can handle a large number of potential moderators in the analysis. Most of these methods optimize treatment-subgroup interactions in general, while QUINT aims at only identifying subgroups that differ in *direction* of the treatment effect (so called *qualitative* treatment-subgroup interactions). Because our interest was in discovering such subgroups, QUINT was used in this study.

The aim of this study was to identify which intervention in the “Be Active & Relax” study [19] - the social environmental or the physical environmental intervention-best suited which group of office workers. It was hypothesized that one or more moderator variables might influence the effect on the primary outcome measure, which was change in NFR from baseline to follow-up.

The research questions of the present study were:

1. Did the effect of the social environmental intervention differ for subgroups of workers and did the effect of the physical environmental intervention differ for subgroups of workers?
2. More specifically, for both interventions, which subgroup of workers showed a positive effect (e.g., a better outcome in NFR) and which subgroup showed a negative effect (e.g., a worse outcome in NFR)?

## Methods

### Study design and study population

For this study, data were used from the “Be Active & Relax” study [19]. From September 2011 to September 2012, 329 office workers participated. They were all recruited at one business location of a Dutch financial service provider. All participants signed an informed consent. This study was a cluster-randomized controlled trial (with three data collection time points) to reduce the NFR in office workers using a physical activity and relaxation program. The study protocol was approved by the Medical Ethics Committee of the VU University Medical Center (Amsterdam, The Netherlands). Additional details regarding the study design and methods of the “Be Active & Relax” study have been published elsewhere [19].

A 2 by 2 full factorial design was applied, resulting in four experimental conditions (see Table 1). Conditions with the social environmental intervention are referred to as SEI^+^ and conditions without as SEI^−^. The same applies to the conditions with the physical environmental intervention (PEI^+^) or without (PEI^−^).

**Table 1:** Two by two factorial design, the social environmental intervention (SEI) and the physical environmental intervention (PEI), including the number of participants per condition

		PEI		Total
		PEI^+^	PEI^−^	
SEI	SEI^+^	63	94	157
	SEI^−^	76	96	172
Total		139	190	329

### Interventions

The delivery modes of the physical activity and relaxation intervention of the “Be Active & Relax” study were systematically developed [19] using a modified version of the intervention mapping protocol [25]. Two types of delivery modes were chosen: a social environmental intervention and a physical environmental intervention.

### Social environmental intervention (SEI)

Four group motivational interviewing sessions were conducted by the team leaders (i.e., after receiving a 2-day training) with the office workers of their own team. The main aim of the sessions was to stimulate physical activity and relaxation by group motivational interviewing sessions (e.g., during session number two, office workers were asked to fill in a worksheet stating their goals and subsequent rewards). The social environmental intervention was supported by a web-based social media platform.

### Physical environmental intervention (PEI)

Based on previous studies [26–32], the aim was to facilitate daily physical activity and relaxation in the work environment, by changing the coffee corners (by adding a bar with bar chairs and a giant wall poster visualizing a relaxing environment), the open office environment (exercise balls and curtains to reduce background noise), the meeting rooms (a standing table and a giant wall poster visualizing a relaxing environment), and the entrance hall (a table tennis table, lounge chairs for informal meetings, and footsteps on the floor to promote stair walking). The interventions PEI and the SEI are described in more detail elsewhere [19].

### Outcome measure

The primary outcome measure of the study was NFR, which was assessed using the NFR after work scale [5]. The questionnaire for the participants was in Dutch. The scale is originally a Dutch scale and is validated for the Dutch population [5]. The scale has good reliability (Cronbach’s alpha = 0.88) and validity. The validity was demonstrated by estimating the associations of NFR with psychosocial risk factors (e.g., emotional load and physical exertion) [5]. Furthermore, it was shown that the NFR has satisfactory test-retest reliability (ICCs 0.68 to 0.80) and is sensitive to detect change in increase in work related fatigue due to the increase in working hours (effect size 0.40) [26]. The NFR after work scale is a sum score of 11 dichotomous items, expressed as a percentage, representing short-term effects of a day at work. Example questions of this scale included: “I find it hard to relax at the end of a working day”, “when I get home from work, I need to be left in peace for a while”. A hundred percent NFR corresponds to a sum score of 11, and represents a very high need for recovery. Our outcome for the analyses is change in NFR from baseline to follow-up (at 12 months). The follow-up score at 12 months was taken to ascertain that the time period was long enough to be able to measure effect of SEI and / or PEI. The follow-up score was subtracted from the baseline score. A positive score means an improvement and represents an absolute reduction in NFR from baseline to 12 months. The minimal relevant difference on the NFR after work scale was set at 12 [26].

### Moderator variables

The “Be Active & Relax” study contained many potential moderator variables. From these variables, we selected the most relevant ones. These were chosen based on the expertise of the researchers, supplemented with moderators that were identified by a literature search. The search was performed in several databases (i.e., PubMed, PsycInfo and Picarta) using the following entries: worksite health promotion, intervention, exercise, workplace, relaxation, and need for recovery. This resulted in variables concerning socio-demographic variables (e.g. age [33], sex, BMI), health and home related variables (e.g. physical activity in free time [34], mental health variables [35], detachment at home) and work related variables (e.g. vitality, team commitment, organizational commitment). For an overview, see Appendix 1. Their univariate effects on NFR were analyzed [16].

### Statistical analyses

#### Introduction of QUINT

Using a new technique like QUINT has some advantages. Compared to the more classical approach of analysis of variance (where the “one size fits all” paradigm is predominate), one advantage of the QUINT method is its ability to identify subgroups that differ in direction of the intervention effect. The results of QUINT give practical indications on how to optimally assign workers to an intervention. Other advantages are that QUINT can handle a large number of moderator variables (in this study, 25 in total) and can easily identify higher order intervention-subgroup interactions, both of which are not possible when using the classical post hoc analysis. Furthermore, the bias-corrected pruning procedure of the method guarantees that no spurious interaction effects will be found.

QUINT is based on a binary recursive partitioning algorithm, which is an algorithm that recursively splits the data in two groups and thus resulting in a binary tree. The partitioning criterion of QUINT maximizes *qualitative* treatment-subgroup interactions. In general, treatment-subgroup interactions imply that the effect of a treatment variable on an outcome variable depends on the levels of (an) other variable(s). These levels define subgroups of persons. In case of *qualitative* treatment-subgroup interactions, the treatment effect differs *in direction* for specific subgroups of persons. This implies that the treatment effect may be positive for one subgroup, and negative for another subgroup. As such, these types of interactions are essential for optimal treatment assignment.

In a QUINT analysis, three types of subgroups are distinguished that are involved in (a) qualitative treatment-subgroup interaction(s). We will elucidate these subgroups for one of our treatment factors, that is, the social environmental intervention with categories SEI^+^ (*n* = 157) and SEI^−^ (*n* = 172). A QUINT analysis partitions the total group of office workers in the following types of subgroups (i.e., partition classes): 1) a group of persons for whom SEI^+^ has on average a better outcome than SEI^−^; 2) a group for whom SEI^−^ has a better outcome than SEI^+^, and 3) a group for whom it does not make any difference. The partitioning criterion of the QUINT algorithm takes into account the difference in treatment outcome and the number of participants in subgroups 1 and 2. The difference in treatment outcome can be specified as a treatment effect size (i.e., Cohen’s *d*) or as a crude difference in means; the corresponding partitioning criterion is called “effect size criterion” or “difference in means criterion”, respectively.

The algorithm of QUINT starts with all persons in one node (the root node). Then it searches for a moderator that optimizes the qualitative treatment-subgroup interaction. The search is performed among all possible moderators, all possible split points on each moderator and all possible assignments of the leaves to the subgroups. The best combination of moderator, split point, and assignment is used to split the total group into two nodes (the “current” leaves). For the next splits, the procedure is repeated in each of the current leaves, and now the combination of moderator, split point, current leaf node, and assignment of the new leaves to the partition classes is chosen that optimizes the qualitative treatment-subgroup interaction.

The splitting process stops if the value of the partitioning criterion cannot be increased anymore. Furthermore, several stopping criteria are taken into account during the splitting process, among which a qualitative interaction condition (i.e., after the first split, the absolute value of the standardized mean difference in treatment outcome in each of the two leaves should exceed a critical minimum value (*d*_min_; the default value is 0.30; this can be seen as a check of whether a qualitative interaction is present in the data), and a minimal sample size per treatment (i.e., in each leaf, a minimum number of participants is in each treatment group for reliable estimation of the treatment means; by default this number is 10 % of the treatment group sample size). After fitting the full tree (with the total number of leaves as *L*), the tree is pruned back using a bootstrap-based bias-correction procedure [22]. This procedure results in bias-corrected criterion values for trees with number of leaves is 1 to *L*. The best pruned tree is the smallest tree that satisfies the one-standard-error rule, that is, it has a bias-corrected criterion value higher or equal to the maximum bias-corrected criterion value minus its standard error. The pruning procedure is described in detail in Appendix 2 of an earlier paper [23]. In an extensive simulation study, it was shown that the inferential errors (Type I and Type II errors) of QUINT were small if the total sample size was higher or equal to 400, *d*_min_ equaled 0.30, and the true treatment effect size was large (i.e., |*d*| ≥ 1). If the sample size was lower or equal to 300, the recovery of tree complexity was acceptable only for smaller trees (i.e., trees with one or two splits) [23].

#### Application of QUINT to the data of the “Be Active & Relax” study

The QUINT analyses were performed using the R-package “quint” [24, 36] in the R version 3.0.2 (R Core Team, 2013). Before starting these analyses, two pre-conditions were tested. The first condition of QUINT is that each person is randomly assigned to an intervention group; however, in the “Be Active & Relax” study, the social environmental intervention was randomized at department (i.e., different service departments of a financial service provider) level, and the physical environmental intervention was stratified on department level, i.e., one stratum with environmental modifications and the other stratum without environmental modifications.

To check if the nesting of office workers within departments could be disregarded, the intra cluster correlation coefficient (ICC) was computed, using improvement in NFR as outcome variable, and the departments as clusters. The ICC was 0.02, indicating that the amount of variance attributed to the departments was very low, and could be neglected. The second condition of QUINT is that the data include only 2 intervention groups, whereas the “Be Active & Relax” study concerned a 2 by 2 design, implying four intervention groups. A re-analysis of the data of the “Be Active & Relax” study using a full factorial analysis of variance (i.e., including main effects of the social environmental intervention and the physical environmental intervention, and the interaction effect between the interventions) with improvement in NFR as outcome variable, revealed that the interaction effect was not significant (nor the two main effects). This result showed that the effect of the social environmental intervention on NFR did not depend on the physical environmental intervention. This allowed us to treat the two factors (i.e., the social environmental and the physical environmental intervention) separately (e.g., analogous to the approach of Raveaud [37]).

To answer the two research questions, two QUINT analyses were performed; one for the social environmental intervention and one for the physical environmental intervention. In both analyses, improvement in NFR was used as the outcome variable and all moderator variables were used as splitting candidates. The number of office workers used in this analysis was 304 (25 of the 329 office workers were deleted due to missing values on one or more moderators). In the final QUINT analyses, the variable “general health” was deleted from the set of moderator variables, because eight office workers had a missing value only on this variable and this variable did not appear to be important (i.e., it was not selected as splitting variable). By deleting “general health”, the number of office workers in the final analysis increased to 312.

As was mentioned before, two partitioning criteria can be used in a QUINT analysis, either the effect size criterion or the difference in means criterion. Our outcome measure can be treated as ordinal (i.e., ratings), as well as numeric (i.e., percentages); therefore, in a first series of QUINT analyses, the effect size criterion was used, and in a second series, the difference in means criterion was used. In this way, it could also be checked whether the solution was stable (i.e., the same) or not. In both series of analyses, a value of 25 was used as minimal sample size per intervention in a leaf, *d*_min_ was set at 0.299, and default values of the weights of the partitioning criterion were used (see supplementary materials in Appendix 1). A large number of bootstrap samples (i.e., *B* = 1000) was used and the one-standard-error pruning rule. Furthermore, independent *t*-tests were performed in each leaf of the pruned tree to test the difference in means of the two groups. It should be noted that the significance level of these *t*-tests is somewhat inflated, due to the data-induced subgroups. Therefore, also the bias-corrected effect sizes in the leaves were estimated using the validation procedure for small data sets (recommended by Dusseldorp & Van Mechelen [23]).

## Results

### Main effects of the delivery modes

The office workers in the social environmental intervention group showed a mean improvement in NFR of 3.82, and those who did not receive the social environmental intervention showed a mean improvement of 1.17 (Table 2); the difference in means was 2.65 (SD = 23.63; effect size *d* = 0.11), and the main effect of the social environmental intervention was not significant (*p* >0.05; indicated by an independent *t*-test). The office workers in the physical environmental intervention group showed a mean improvement in NFR of 4.59, and those who did not receive the physical environmental intervention showed a mean increase of 0.85 (Table 2); the difference in means was 3.75 (SD = 23.60; effect size *d* = 0.16), and the main effect of the physical environmental intervention was not significant (*p* > 0.05; indicated by an independent t-test). The mean values (or percentages) on all included moderator variables for the groups with and without each delivery mode are shown in Table 2.

**Table 2:** Descriptive statistics for all variables involved in re-analyses of data from the “Be Active & Relax” study. The potential moderators were all measured at baseline (i.e., before receiving a physical activity and relaxation program). The statistics are given for both delivery modes: the social intervention and the physical environmental intervention (*N* = 312)

			Delivery mode: social environmental intervention				Delivery mode: physical environmental intervention			
			Yes *n* = 149		No *n* = 163		Yes *n* = 132		No *n* = 180	
Variable	Range		Mean	(SD)	Mean	(SD)	Mean	(SD)	Mean	(SD)
Outcome										
Improv. in need for recovery	−100.0	81.82	3.82	(25.32)	1.17	(21.98)	4.59	(23.38)	0.85	(23.75)
Potential moderators										
Need for recovery at baseline	0.00	100.00	30.42	(28.90)	30.36	(28.88)	32.18	(30.28)	29.08	(27.75)
Age (in years)	19.00	63.00	42.46	(10.05)	41.77	(9.89)	41.63	(10.39)	42.45	(9.63)
Sex (male vs. female)	0	1	0.62	(0.48)	0.63	(0.48)	0.62	(0.49)	0.63	(0.48)
Level of education	1.00	3.00	2.29	(0.86)	2.41	(0.77)	2.46	(0.76)	2.27	(0.84)
Cohabiting (yes vs. no)	0	1	0.75	(0.43)	0.77	(0.42)	0.8	(0.40)	0.74	(0.44)
Mother country (Neth. vs. other)	0	1	0.93	(0.26)	0.91	(0.29)	0.93	(0.25)	0.91	(0.29)
Body Mass Index	17.10	39.19	25.18	(4.35)	24.87	(3.74)	24.61	(3.56)	25.31	(4.34)
Mental Health	2.00	6.00	4.5	(0.72)	4.51	(0.73)	4.42	(0.69)	4.57	(0.74)
Detachment at home	1.00	7.00	4.76	(1.33)	4.9	(1.35)	4.8	(1.39)	4.86	(1.31)
Relaxation at home	2.00	7.00	5.16	(1.02)	5.25	(1.11)	5.05	(1.07)	5.33	(1.05)
Physical activity (in MET-min.)	375	29610	7527	(4234)	7521	(3937)	7066	(4018)	7860	(4095)
Vitality	2.00	7.00	5.00	(0.96)	5.06	(1.00)	4.92	(0.97)	5.11	(0.98)
Team commitment	1.00	5.00	4.07	(0.65)	4.14	(0.68)	3.99	(0.64)	4.19	(0.67)
Organizational commitment	2.57	5.00	4.00	(0.47)	4.08	(0.44)	3.97	(0.44)	4.09	(0.46)
Supervisor support	1.00	4.00	2.87	(0.51	2.89	(0.48)	2.86	(0.53)	2.89	(0.47)
Colleague support	2.00	4.00	3.09	(0.38)	3.09	(0.37)	3.05	(0.37)	3.12	(0.37)
Job demands	1.50	4.00	2.82	(0.49)	2.71	(0.40)	2.78	(0.46)	2.75	(0.44)
Decision authority	1.00	4.00	2.98	(0.53)	2.99	(0.54)	2.98	(0.56)	2.99	(0.52)
Job insecurity	1.00	3.00	1.55	(0.39)	1.65	(0.48)	1.58	(0.42)	1.62	(0.45)
Skill discretion	1.83	4.00	3.03	(0.37)	3.09	(0.37)	3.1	(0.39)	3.03	(0.35)
Working overtime (in hrs. p. wk.)	0.00	40.00	2.85	(6.05)	3.19	(7.78)	2.74	(6.70)	3.25	(7.22)
Detachment at work	1.00	7.00	3.48	(1.39)	3.54	(1.34)	3.46	(1.30)	3.54	(1.41)
Relaxation at work	1.00	7.00	3.53	(1.25)	3.69	(1.31)	3.45	(1.19)	3.74	(1.33)
Walking during lunch	1	5	2.78	(1.45)	2.94	(1.47)	2.86	(1.39)	2.87	(1.52)
Active during lunch	1	4	1.92	(1.04)	1.91	(1.04)	1.83	(0.98)	1.97	(1.08)

### Results of the QUINT analyses

**Fig. 1:**
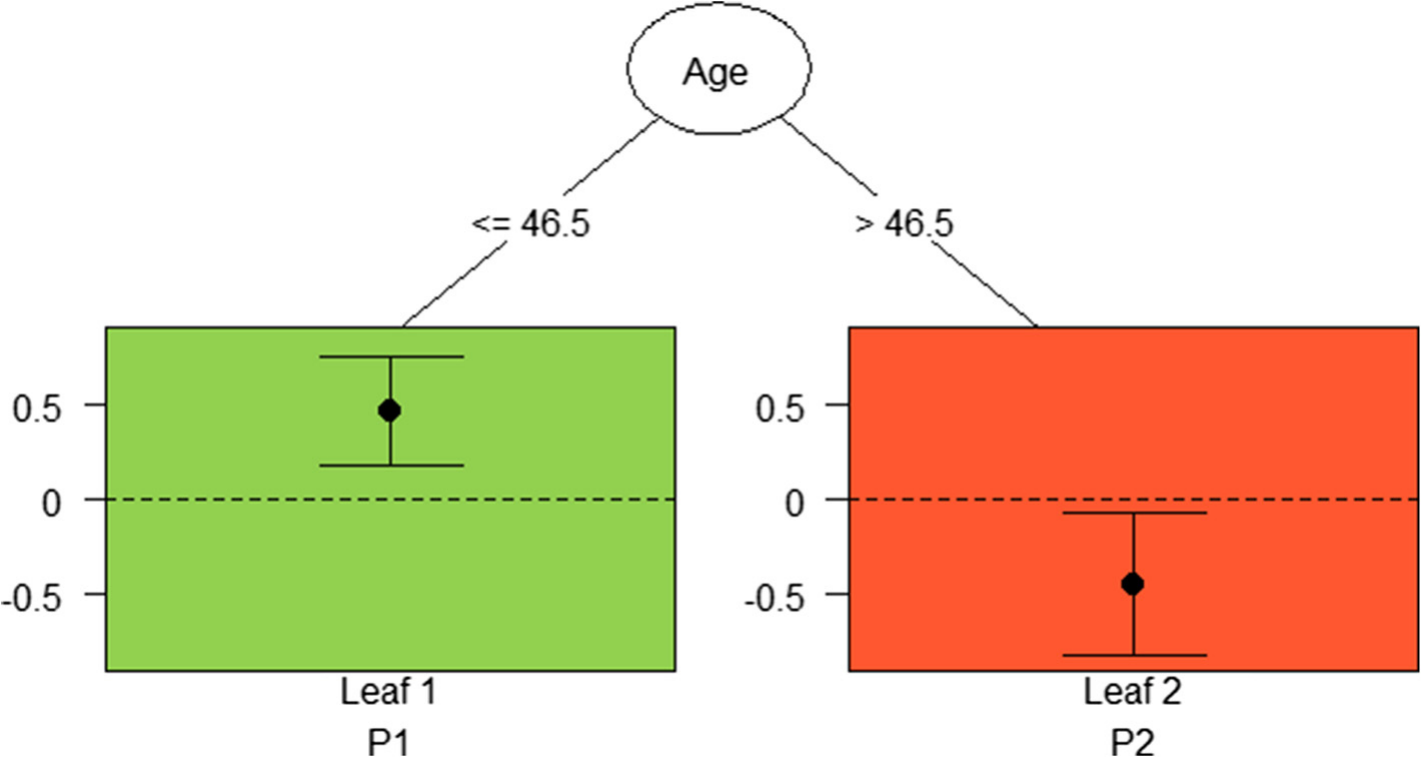
Pruned tree for social environmental intervention with moderator variable “Age” with two leaves *(File ‘Fig.* 1 *pruned.png’).* Legend: Pruned tree involving the variable Age and a split point of 46.5 years; the effect sizes *d* are expressed as the standardized mean difference between the group with the social environmental intervention (SEI^+^) and the group without (SEI^−^); For the leaf assigned to P1 (i.e., the left green leaf) the effect size *d* is positive, while for the leaf assigned to P2, the effect size *d* is negative

**Fig. 2:**
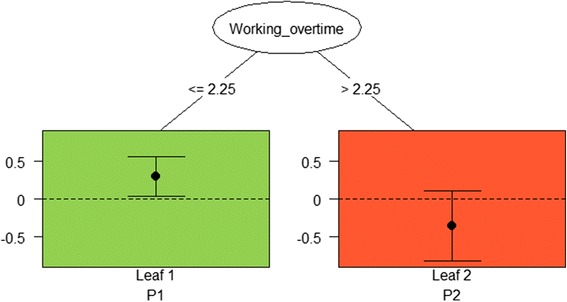
Pruned tree for physical environmental intervention with moderator variable “Working overtime” with two leaves *(File ‘Fig.* 2 *pruned.png’).* Legend: Pruned tree involving the variable Working overtime and a split point at 2.25 h indicating that office workers who worked fewer hours overtime (≤ 2.25) had a better outcome with the physical environmental intervention than without the physical environmental intervention (Leaf 1) and office workers who worked more hours overtime (> 2.25) had a worse outcome with the physical environmental intervention than without (Leaf 2)

The first series of QUINT analyses resulted in the same full trees as the second series of analyses. However, the pruning results were different: the pruned trees from the effect size criterion were smaller than those from the difference in mean criterion. Because the sample size of this study was relatively small for a QUINT analysis (i.e., around 300), the smaller pruned trees were preferred to the larger ones to take a conservative approach. Therefore, the results of the first series of analysis are described below and presented in Figs. 1 and 2, those from the second series are presented in Appendix 2.

The result for the social environmental intervention was a pruned tree with two leaves. The split of the tree (Fig. 1) involved the variable “Age” and a split point of 46.5 years. It should be noted that in the leaves of Fig. 1, the effect sizes *d* are expressed as the standardized mean difference between the group with the social environmental intervention (SEI^+^) and the group without (SEI^−^). As a consequence, for the leaf assigned to P1 (i.e., the left green leaf), the effect size *d* is positive, while for the leaf assigned to P2, the effect size *d* is negative. The results indicated that younger office workers (≤46.5 years) who received the social environmental intervention had a better outcome in NFR than younger office workers who did not receive the social environmental intervention (Leaf 1 in Fig. 1; difference in means = 10.52; 95 % CI: 4.12, 16.92); bias-corrected effect size *d* = 0.32, Table 3).

**Table 3:** Descriptive statistics in the leaves of the quint results for the social environmental intervention (SEI; Fig. 1) and the physical environmental intervention (PEI; Fig. 2). The mean values and standard deviations on improvement in Need for Recovery (NFR) are displayed (i.e., a higher score reflects a larger reduction in NFR from baseline to 12 month follow-up), and the treatment outcome differences

	*n*	Mean	SD	*n*	Mean	SD	Difference in means (95 % CI)	Bias-corrected effect size *d*
Fig. 1	SEI^+^			SEI^−^				
Leaf 1	90	8.29	22.27	107	−2.23	23.20	10.52 (4.12, 16.92)*	0.32
Leaf 2	59	−3.00	28.22	56	7.66	17.89	−10.65 (−19.35, -1.96)*	−0.22
Fig. 2	PEI^+^			PEI^−^				
Leaf 1	103	6.15	23.90	128	−1.25	25.39	7.40 (0.99, 13.81)*	0.13
Leaf 2	29	−0.94	20.90	52	6.01	18.35	−6.95 (−16.26, 2.36)	−0.08

*Note. CI* confidence interval; *p < .05, estimated by independent t-test

Furthermore, the results showed that older office workers (> 46.5 years) who received the social environmental intervention had a worse outcome than older office workers who did not receive the social environmental intervention (Leaf 2 in Fig. 1; difference in means = -10.65; 95 % CI: −19.35, −1.96); bias-corrected effect size *d* = -0.22, Table 3).

The result for the physical environmental intervention was also a pruned tree with two leaves. The split of the tree involved the variable “Working overtime” and a split point at 2.25 h (Fig. 2). The results indicated that office workers who worked fewer hours overtime (≤ 2.25) had a better outcome with the physical environmental intervention than without the physical environmental intervention (Leaf 1 in Fig. 2; difference in means = 7.40; 95 % CI: 0.99, 13.81); bias-corrected effect size *d* = 0.13). Furthermore, the results showed that office workers who worked more hours overtime (> 2.25) had a worse outcome with the physical environmental intervention than without, but this effect was not significant (Leaf 2 in Fig. 2; difference in means = −6.95; 95 % CI: −16.26, 2.36); bias-corrected effect size *d* = −0.08, Table 3).

## Discussion

This study examined which subgroups benefit more from which delivery mode of a physical activity and relaxation program during work on NFR. The results from the QUINT analysis suggest that a social environmental intervention might be more beneficial for younger workers, and a physical environmental intervention might be more beneficial for employees with a only few hours overtime.

### Comparison with other studies

#### Observed change in NFR

Although the “Be Active & Relax” study demonstrated no significant effect on the main outcome NFR [16], differential treatment efficacy was found in our study. The changes in NFR found from baseline to 12-month follow-up in the intervention subgroups (i.e., 8.29 for the social environmental and 6.15 for the physical environmental intervention) were somewhat lower than the change found by De Croon et al. [26], that is, a difference in NFR of 12.6. However, the latter difference concerned an *increase* of NFR in a non-experimental design and was determined over a longer period (2 years).

#### Age

Several studies found age to be an important factor in the effectiveness of worksite health programs [38, 39]. The review by Rongen et al. [38] demonstrated that the effectiveness of workplace health promotion programs was larger in younger populations (mean age of <40, with a difference in effect size of -0.17; 95 % CI -0.23, −0.17). Specifically, 18 studies were compared that had studied an intervention aimed at a healthy lifestyle (such as physical activity, healthy nutrition, weight loss, or smoking cessation). The delivery mode varied from a tailored fitness program to counseling sessions focusing on physical activity and nutrition. The authors of this review advised to target specific age populations in worksite interventions. The study also found workplace health promotion programs to be more effective in white collar workers.

Furthermore, Hughes et al. [39] found an individualized program to be more effective than a program where older university workers could choose health modules on their own, and received generic health e-mail tips. Participants from the individualized intervention reported a borderline significant reduction in percentage energy from fat at 6 months (*p* = .063) and a significant reduction at 12 months (*p* < .05) and the group reported significantly more minutes of moderate physical activity than did controls at 6 and 12 months (*p* < .05). At last, a significant decline in waist circumference at 6 months was achieved by the intervention group that was maintained at 12 months (*p* < .05). Stress and smoking did not change significantly. These results underline the claims for individualizing the approach for older office workers.

The social environmental intervention had a negative effect on older workers. It could be that younger employees had a more positive attitude toward the social environmental intervention than older employees.

Furthermore, there is some evidence that being a good role model as a team leader in terms of healthy behaviors may be important for those offering the social environmental intervention to support physical activity and relaxation [16]. In addition, other characteristics of the team leaders (i.e., age, gender, ethnicity, or level of education) might have influenced the effectiveness of the social environmental intervention.

Finally, the older participants in our study (> 46.5 years old) may have been healthier than the office workers who have left the job (healthy worker effect). The baseline NFR score of the older participants was relatively low (i.e., 28.0) and did not differ significantly from the younger ones. This finding is in contrast with the studies of Kiss et al. [40] and Mohren et al. [41], who found that older workers had a higher NFR than younger workers. Kiss et al. [40] found that the mean recovery score (40.9) was significantly higher in the group of the older public sector workers as compared to the mean score (33.6) of younger workers (*p* < 0.05). Mohren et al. [41] studied day workers and discovered that the highest levels of NFR were observed in the age group of 46–55 years. The relative risk for developing elevated NFR was highest in the age groups 36–45 years (RR 1.30; 1.07–1.58) and 46–55 years (RR 1.25; 1.03–1.52) in men and 46–55 years (RR 1.36; 1.04–1.77) in women when compared to the reference group.

#### Working overtime

Working overtime means that a person is putting in more hours than agreed upon in his or her contract. Although the threshold for working overtime to benefit from environmental modifications in this study is rather low (on average 2.25 h per week), underlying mechanisms may explain why office workers who work overtime do not benefit fully from the delivery mode environmental modifications.

Research indicates two possible mechanisms for overtime work causing an increase in stress levels [18]. The first is that high levels of overtime may lead to lack of recovery because of the shorter periods of rest between working days. This can affect recovery time and increase the exposure time to work stress, in turn resulting in adverse health and well-being. The second mechanism affects health indirectly and concerns behavioral decisions and habits (i.e., lifestyle behaviors) of workers. Taris et al. found overtime to lead to lower levels of physical activity and intake of fruits and vegetables in full-time workers. Working overtime simply reduces the amount of time and energy available to be physically active [18].

It could be that the participants in this study who worked more hours overtime did not make as much use of the environmental modifications as the participants who did not work overtime because of time constraints. As a result, working overtime may have interfered with fully benefitting from the environmental modifications. Office workers who work overtime on a regular basis probably need a different intervention to change their lifestyle and make them more physically active. It may be advisable for organizations to pay attention to the work process when working overtime is structural. Office workers who work overtime structurally may be a vulnerable group. Not only do they have stress as a result of working extra hours, but they may also be less susceptible for interventions aimed at physical activity and relaxation.

Looking at the evaluation of the “Be Active & Relax” study [42], it is remarkable that the element in the environmental modifications that was most physically activating (e.g. table tennis table) was not used much. It was shown that the percentage that used the table tennis table at least once on a scale from 0 (never) to 5 (always) was on average 17 % of the 35 participants that were evaluated.

#### Limitations of this study

There are some limitations of this study. First, the compliance of the sample. Not all participants in the “Be Active & Relax” study fully participated in the social and in the physical environmental interventions [42]. The reach (% of participants that attended at least one sessions or used at least one element) for the social and physical environmental intervention ranged from 45 to 76 % [42]. A barrier for not attending the social environmental intervention sessions were, for example, having a holiday. An important barrier for the participants for not using the physically activating and relaxing elements in the physical environmental intervention was office workers indicating that they did not have enough time. Although the level of reach is comparable to other worksite health programs (mostly below 50 % [43]); this could have underestimated the results in the QUINT analysis.

Although no significant effect was found on the main outcome measure of NFR in the study by Coffeng et al. [16], secondary outcome measures did differ significantly. In the combined environmental intervention group (*n* = 92), exhaustion and vigorous physical activities decreased significantly, and small breaks at work and active commuting increased significantly compared to the control group. The social environmental intervention group (*n* = 118) showed a significant reduction in exhaustion, and sedentary behavior at work, and a significant increase in small breaks at work and leisure activities compared to the control group. In the physical environmental intervention group (*n* = 96), stair climbing at work and active commuting significantly increased, and sedentary behavior at work decreased significantly compared to the control group.

However, generalizability of recommending the social environmental intervention for younger office workers and using the physical environmental intervention for office workers who do not work overtime should be interpreted cautiously.

A second limitation of the study was that the existence of possible subgroups that benefit more from the combination of the social and physical environmental intervention was not explored due to sample size restrictions. Although the statistical analyses did not show the need for doing this (due to a non-significant interaction effect between the social environmental intervention and the physical environmental intervention), it cannot be ruled out that subgroups exist that might benefit especially from the combined intervention.

The generalizability of the interventions to other work environments is questionable, because the interventions were specifically tailored to the target population by using intervention mapping. Furthermore, our study population consisted of 60 % males and 57 % was highly educated, which does not represent the general Dutch working population. This was due to the fact that the financial service provider’s workforce involves mainly highly educated, male and white collar employees.

Finally, the “Be Active & Relax” study was not a full RCT. The social environmental intervention group was randomized, but the physical environmental intervention was not (for details, see [19]). Therefore, cautiousness is needed in the interpretation of the results of the physical environmental intervention; the beneficial effect for office workers with a few hours overtime might not have been *caused* by the intervention, due to the non-random assignment.

#### Methodological issues

In the present study, the QUINT analyses were performed using the effect size criterion and the difference in means criterion. Both analyses led to the same full trees, but different pruned trees. Due to the relatively low sample size (*N* = 312) for a QUINT analysis, we took a conservative approach and preferred the smaller trees (for detecting complex interactions, *N* ≥ 400 is recommended [23]). In future research, larger sample sizes are recommended to confirm our hypotheses. The software of QUINT allows the user to choose between several parameters, for example, the minimum sample size per intervention condition in a leaf and the total number of bootstrap samples used for the pruning procedure. In the present study, we found that results were more stable using a minimum intervention sample size of 25 (instead of the default value of 10 %), and a total number of bootstrap samples of 1000 (instead of the default value of 25).

#### Implications for research and practice

The implications of the results of our study are threefold. Firstly, the results suggest that a “one size fits all” approach does not hold for the worksite health program “Be Active and Relax”. For other worksite health programs, this commonly used approach may also not work. Instead, we recommend the developers of such programs to carefully consider possibilities to tailor the program to specific subgroups. Secondly, the study shows that an advanced exploratory analyses method, like QUINT, is able to indicate for which subgroups of workers a worksite health program is beneficial and for which subgroups it is not. And thirdly, the results suggest that group motivational interviewing by team leaders is not an appropriate strategy for older workers to reduce their need for recovery. In addition, changing the work environment to facilitate daily physical activity and relaxation at the worksite is not appropriate for workers who work more hours overtime. More research is needed to investigate which type of health program would work for these specific subgroups of office workers. Based on the results, we advise to take age and working overtime into account when developing a health program for office workers, and also when designing health intervention evaluation studies.

## Conclusions

This study was conducted to explore whether subgroups may benefit more from a social or a physical environmental intervention to reduce the NFR by using the QUINT method.

Specifically, the results suggest possible roles for age and working overtime: younger workers benefitted (in terms of a reduced need for recovery) from a social environmental intervention and employees who worked fewer hours overtime benefitted from a physical environmental intervention. It is recommended to incorporate age and working overtime as stratification variables in future research into the need for recovery among office workers to confirm these results, and to tailor the interventions to specific groups.

## Figures and Tables

**Fig. 3:**
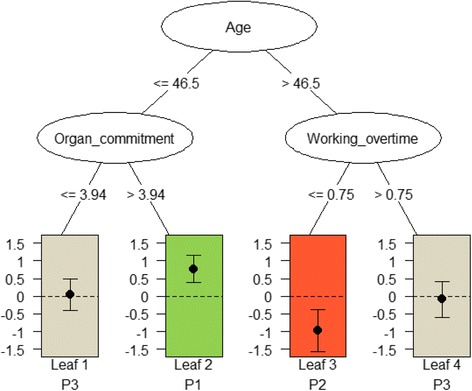
QUINT solution for the delivery mode “social environmental intervention”, using the Difference in means criterion

**Fig. 4:**
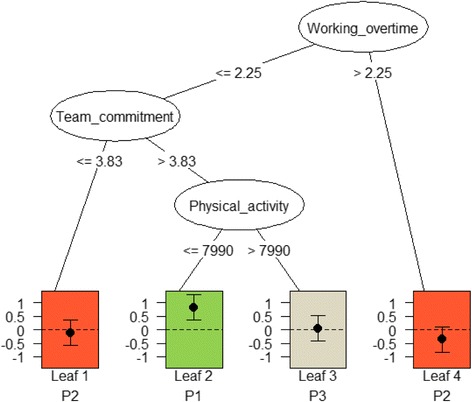
QUINT solution for the delivery mode “physical environmental intervention”, using the Difference in means criterion

## Appendix 1

**Relevant moderator variables measured in the “Be Active & Relax” Project**.

**Table 4:** The potential moderators were all measured at baseline (i.e., before receiving a physical activity and relaxation program) and are divided into background variables, health and home related variables and work related variables

	# it	α or *r*	Example of an item with answer categories and score range	Questionnaire	Studies indicating a possible modifying effect
Background					
Age	1	N/A	“What is your date of birth?”	N/A	Steenstra (2009) [33]; Demou (2012) [48], Bauman (2012) [49]
Sex	1	N/A	“What is your gender?”; 2 categories “man” or “woman”	N/A	Demou (2012) [48], Bauman (2012) [49]
Level of education	1	N/A	“What is the highest level of education you have completed?; 8-point scale from 1”no education” to 8 “university degree”	N/A	
Cohabiting	1	N/A	“What is your current marital status?”; 6 categories (such as married, living with another, etc)	N/A	
Mother country	1	N/A	“In what country were you born?”; 2 categories: the Netherlands or other	N/A	
Health/home					
BMI	2	N/A	“What is your weight?” en “How tall are you?”	N/A	Colkesen (2011) [34]
General health	1	N/A	“In general, how would you say your health is?”; 5-point scale from 1 “excellent” to 5 “poor”	Rand-36; Van der Zee (1993) [44]	Demou (2012) [48], Colkesen (2011) [34], Bauman (2012) [49]
Mental health	9	α = 0.90	“Did you feel full of pep?”; 6-point scale from 1 “none of the time” to 6 “all of the time”	Rand-36; Van der Zee (1993) [44]	Demou (2012) [48]; Dishman (2010) [35]
Detachment at home	4	α = 0.94	“After work, I don’t think about work at all”; 7-point scale from 1 “never” to 7 “almost always”	The recovery experience questionnaire; Sonnentag (2007) [14] and De Bloom (2011) [45]	
Physical activity	7	r = 0,97	“Do you walk/cycle to work? On how many days per week? What is the average time it takes? Rate the intensity: light, moderate, vigorous”	Short Questionnaire to Assess Health Enhancing Physical Activity (SQUASH); Wendel-Vos (2003) [46]	Colkesen (2011) [34]
Relaxation at home	4	α = 0.92	“After work, I do relaxing things”; 7-point scale from 1 “never” to 7 “almost always”	The recovery experience questionnaire; Sonnentag (2007) [14] and De Bloom (2011) [45]	
Work related					
Vitality	5	α = 0.83	“At my work I feel bursting with energy”; 6-point scale from 1 “never” to 6 “always”	Part of Utrecht Work engagement Scale (UWES); Demerouti (2001)	Strijk (2012) [17]
Team commitment	3	α = 0.82	“What I feel for this team – I am proud”; 5-point scale from 1 “totally agree” to 5 “totally disagree”		
Organizational commitment	8	α = 0.79	“I find it important that my job go’s well”; 5-point scale from 1 “strongly agree” to 5 “strongly disagree”		Meyer (2002) [50]; Lohela (2009) [51]
Supervisor support	4	α = 0.78	“My supervisor is concerned about the welfare of those under him”; 4-point scale from 1 “strongly agree” to 4 “strongly disagree”	Dutch version of the Job Content Questionnaire Karasek (1998) [47]	Choi (2011) [52]
Colleague support	4	α = 0.76	“People I work with are competent in doing their jobs”; 4-point scale from 1 “strongly agree” to 4 “strongly disagree”	Dutch version of the Job Content Questionnaire Karasek (1998) [47]	Choi (2011) [52]
Job demands	5	α =0.70	“My job requires working very fast”; 4-point scale from 1 “strongly agree” to 4 “strongly disagree ”	Dutch version of the Job Content Questionnaire Karasek (1998) [47]	Lohela (2009) [51], Van Laethem (2013) [53]
Decision authority	3	α =0.68	“My job allows me to make a lot of decisions on my own”; 4-point scale from 1 “ strongly agree” to 4 “strongly disagree ”	Dutch version of the Job Content Questionnaire Karasek (1998) [47]	Mullan (1985) [54]
Skill discretion	6	α =0.67	“My job requires that I learn new things”; 4-point scale from 1 “strongly agree” to 4 “strongly disagree ”	Dutch version of the Job Content Questionnaire Karasek (1998) [47]	(WEBA)
Job insecurity	3	α =0.68	“During the past year were you in a situation where you faced job loss or layoff?”; 3-point scale from 1 “no” to “actually layed off”	Dutch version of the Job Content Questionnaire Karasek (1998) [47]	Karlsson (2010) [55], Lohela (2009) [51]
Working overtime	1	N/A	“On average, how many hours of overtime do you put in per week?”		Taris (2011) [18]
Detachment at work	4	α = 0.93	“During a break at work I forget about my work”; 7-point scale from 1 “never” to 7 “almost always”	Adapted question (at work was added) of “The recovery experience questionnaire”; Sonnentag (2007) [14] and De Bloom (2011) [45]	
Relaxation at work	4	α = 0.88	“During a break at work I use the time to relax”; 7-point scale from 1 “never” to 7 “almost always”	Adapted question (at work was added) of “The recovery experience questionnaire”; Sonnentag (2007) [14] and De Bloom (2011) [45]	

Notes: *# it* number of items, *α* Cronbach’s α, *r* Pearson’s correlation coefficient, *N/A* not applicable

## Appendix 2

**Results of the second series of analyses**

The full tree that resulted from the QUINT analysis for the social environmental intervention and for the physical environmental intervention using the Difference in means criterion was equal to the full trees using the Effect size criterion (the results from the latter were described in the Results section). However, the pruning procedure resulted in larger trees for both the social environmental intervention and the physical environmental intervention, that is, trees with four leaves (see Figs. 3 and 4). For the social environmental intervention, additional splitting variables were Organization commitment and Working overtime (Fig. 3).

The results showed that the social intervention was especially effective for the younger employees with a high Organization commitment (Leaf 2 in Fig. 3; bias-corrected effects were *d* = 0.38 and difference in means = 10.40). Furthermore the social environmental intervention was counter-effective for older persons working less than one hour of overtime (Leaf 3 in Fig. 3; *d* = -0.97; bias-corrected effects were *d* = −0.67; difference in means = −16.29).

For the physical environmental intervention, additional splitting variables were Team commitment and Physical activity (Fig. 4). The results showed that the physical environmental intervention was especially effective for the employees working less hours overtime with a high Team commitment and a lower level of Physical activity (Leaf 2; bias-corrected effects were *d* = 0.42 and difference in means = 11.40).
